# Leveraging data augmentation for machine learning models in predicting depression and anxiety using the Revised Child Anxiety and Depression Scale clinical reports

**DOI:** 10.3389/fpsyt.2025.1672178

**Published:** 2025-11-27

**Authors:** Saleha Noor, Zamir Hussain, Qurrat Ulain Hamdan, Mehwish Zaman, Rehan Zafar Paracha, Syeda Aneela Zahra Shamsi

**Affiliations:** 1 School of Interdisciplinary Engineering and Sciences (SINES), National University of Sciences & Technology (NUST), Islamabad, Pakistan; 2 Institute of Psychiatry, Rawalpindi Medical University, Rawalpindi, Pakistan; 3 Department of Statistical Science, University of Padua, Padova, Italy; 4 Riphah Institute of Pharmaceutical Sciences, Riphah International University, Islamabad, Pakistan; 5 National Institute of Health, Chak Shahzad, Islamabad, Pakistan

**Keywords:** Revised Child Anxiety and Depression Scale (RCADS), machine learning algorithms, depression, anxiety, data augmentation

## Abstract

**Objective:**

An estimated 15 million people are affected by depression and anxiety in Pakistan. However, there are relatively few government mental health facilities and certified psychiatrists. This highlights the need for efficient assessments to implement intervention strategies and address these challenges. This study aims to utilize machine learning with RCADS to maximize the use of current healthcare resources and facilitate depression and anxiety screening.

**Methods:**

The dataset include 138 cases, with 89 retained after cleaning along 47 RCADS-items as features. Based on RCADS-47 T-scores, cases were classified as normal, borderline and clinical, with 7% in the borderline, 55% in normal and 38% in clinical range. Three feature selection methods - the Chi-square test of independence, Spearman’s correlation, and Random Forest-Recursive Feature Elimination were performed. Data augmentation was done using the probability distribution of the existing data to generate hybrid-synthetic correlated discrete multinomial variants of each item of RCADS. Six commonly employed ML algorithms, Decision Tree, Random Forest, Support Vector Machine, Logistic Regression, Naive Bayes, and K-Nearest Neighbor, were trained on the original dataset and the top three best models were then evaluated on augmented datasets and the best among them, further validated on external dataset.

**Results:**

Item 05 of the RCADS has a weak correlation with the evaluation of depression and anxiety in the study population. Data augmented to forth time its original size was determined to be the optimal ratio for our dataset as Random Forest yielded the best overall results with up to 81% macro average accuracy, precision, recall and F1 score when tested on this data.

**Conclusion:**

The findings suggest that the Random Forest algorithm using 46 features suits the data well and has the potential to be further developed as a decision support system for the concerned professionals and improve the usual way of screening anxiety and depression in children and adolescents.

## 1 Introduction

Adolescence is a transitional stage with a maelstrom of change that makes young people more susceptible to mental health illnesses such as anxiety, mood disorders, eating disorders, and personality disorders (1). The most prevalent forms of mental health problems in children and adolescents include psychological distress such as depression and anxiety (2, 3). The rates range from 11% to 25% globally for anxiety disorders and from 3% to 8% for depressive disorders (4, 5). Studies have shown that untreated anxiety and depression may have negative effects and cause other issues later in life, such as substance misuse or dependency, suicidal thoughts, poor academic performance, and unemployment (6–9). The Diagnostic and Statistical Manual of Mental Disorders (DSM-5) defines anxiety as worrying about a potential threat, whereas fear is defined as an emotional response to genuine or impending harm. Depressive disorder, on the other hand, is an umbrella term for illnesses that cause continuous feelings of sadness and accompanying changes that greatly impair one’s capacity to function (10). Among the depressive disorders is Major Depressive Disorder (MDD), which was previously placed in the “Mood Disorders” chapter of DSM-IV, and is now located in the “Depressive Disorder” section of DSM-5 (11).

Given the qualitative nature of anxiety and depression, assessments often consist of personal interviews or questionnaires. Various tools have been developed to evaluate anxiety and depression in children and adolescents such as the Fear Survey Schedule for Children-Revised (12), Spielberger State-Trait Anxiety Inventory for Children (13), and the Revised Children’s Manifest Anxiety Scale (14). The Revised Child Anxiety and Depression Scale (RCADS), which is a revised version of the Spence Children’s Anxiety Scale (15) is another such tool. It provides scales that index the key characteristics of five common DSM-IV anxiety disorders, namely separation anxiety disorder, social phobia, generalized anxiety disorder, panic disorder, obsessive-compulsive disorder, and low mood (major depressive disorder). It is a freely available 47-item self-report measure used to evaluate children’s symptoms that align with major depressive and anxiety disorders in the DSM-IV. Self-report measures are essential for assessing these disorders in children because they offer personal accounts of their experiences that cannot be obtained from other sources. The RCADS has depression measures in addition to scales covering the most prevalent anxiety disorders in young people. Considering how frequently anxiety and depression co-occur in young people, this is advantageous in comparison to many other self-report measures that evaluate just anxiety. Additionally, a scale for obsessive-compulsive disorder (OCD), which is underdiagnosed, undertreated, and under-detected in children and adolescents, is also included in the RCADS. The RCADS has also demonstrated promising psychometric properties across multiple countries (16). RCADS was created by American researchers and was first tested on American individuals. Since then, it has been validated in different populations such as Australia (17), Denmark (18), Netherlands (19), Turkey (20), Ireland (21), El Salvador (22), and The United Kingdom (23). Furthermore, it has shown good psychometric properties in both meta-analyses and cross-cultural studies (16, 24). Even though the RCADS has been extensively applied to measure anxiety and depressive symptoms in children, there have been a number of psychometric and contextual questions of concern as highlighted in some studies. Although most of the studies reproduce the six-factor structure of the subscales of anxiety and depression, other studies have also reported that the factor loadings of some subscales are weak in some cultural or ethnic subgroups (e.g., obsessive-compulsive, major depression disorder) (19). In addition, measurement invariance (i.e. whether items are similar in different age groups, gender or culture) is not necessarily entirely established. As an example, cross-cultural research has found that a small set of RCADS items exhibit a different item functioning (DIF) among countries, i.e. the item may not be interpreted or reacted to in similar ways (25). The subscale of depression in particular has demonstrated relatively poor structural validity and test retest reliability when compared to the subscales of anxiety (especially in shorter versions, like the RCADS-25 and RCADS-20) (26). In a similar manner research involving autistic youth has also established that total anxiety scores exhibit high reliability, but depression subscales can exhibit moderate or doubtful consistency between parent and child ratings (27). Last but not least, although the RCADS is translated and tested across various languages, cross-cultural studies have noted that cultural norms and stigma can affect reporting of symptoms, some of the items are not effective at reflecting culturally specific manifestations of anxiety and depression, particularly in low- and middle-income nations (25, 28). Though RCADS-47 is a well-validated scale to measure anxiety, and depressive symptoms in youth, the majority of research on this scale has concentrated on psychometric validation and intercultural measurement as opposed to predictive machine-learning uses of the scale, with item-level RCADS responses as features. The existing literature lacks studies that incorporate RCADS into ML screening pipelines and even the instances of RCADS being present in ML data are typically the use of an outcome label or in combination with other modalities instead of making it the primary input (29, 30). To the best of our knowledge there is no other published study that has integrated RCADS-47 item responses and supervised ML to enhance screening in an LMIC context, including Pakistan. Since sociodemographic factors of study populations are reported as key influencers in the development of early-onset psychological disorders (31), it is necessary to assess the RCADS using local datasets where anxiety and depression prevalence is different. Although native measures like the Aga Khan University Anxiety and Depression Scale (AKUADS) and the Pakistan Anxiety and Depression Questionnaire (PADQ) have been effective in local screening, they are typically shorter and lump anxiety and depression together and are usually less specific to subtypes of anxiety. RCADS-47, in turn, also has several anxiety disorder subscales (separation anxiety, social phobia, panic disorder, generalized anxiety, obsessive-compulsive disorder) as well as a separate depression scale, and thus can screen in a finer-grained way, which is particularly likely to be critical in children whose manifestations of anxiety and depression are often more subtype-specific. Besides, the strong psychometric support of RCADS in large multi-ethnic samples (e.g., the Dutch urban children sample that has shown good factor structure and reliability) indicates that it will be more generalizable to different populations. Significantly to the Pakistani children, currently in use screening methods have limitations: they do not demonstrate measurement invariance, somatic symptoms are not adequately covered, and screening methods fail to distinguish between the depressive and anxiety subtypes. Appropriately translated/adapted and validated RCADS-47, therefore, provides a more complete, reliable, and informative screening tool to use in this population.

Even though the mental health of children is becoming a global priority, there is limited research on the subject in Pakistan. Approximately 15 million people are affected by mental health issues, and despite this, there are relatively few government mental health facilities and roughly 400 certified psychiatrists, the majority of whom are located in urban areas (32). Pakistan has a population of 241.49 million (33) and about half of this population is under the age of 18 (34) but no empirical statistics for children and adolescents have been recorded on a national level. Nonetheless, studies and surveys with small sample numbers show that there is a burden associated with early onset mental health problems. A survey conducted in Rawalpindi on 1,124 youth revealed that 17.2% and 21.4% of them were estimated to be suffering from anxiety and depression, respectively (35). A study conducted in Karachi on 1,470 individuals between the ages of 11 and 17 found that around 20% of the participants had serious emotional and behavioral issues. Similarly, a survey conducted on 640 teenagers estimated that 34% of the participants had atypical social and emotional behavior (36, 37). Regardless of the given estimates, resources for addressing mental health disorders are insufficient for their severity. When it comes to mental health concerns, the majority of the community appears to be in denial. Individuals are reluctant to disclose that they suffer from mental health issues because these subjects are hardly discussed (38). The general public is unaware of mental illnesses and the small percentage that is informed is unaware of the therapies available for them. These reasons lead to untreated mental disorders. The stigma associated with mental health is a serious obstacle to research initiatives. The perception of mental illness as a personal shortcoming deters people from communicating and getting care (32). This reluctance to provide information limits the use of approaches that rely on self-reported data. The negative perception makes it difficult to gather large samples of data for mental health research. Since machine learning algorithms perform better when trained on huge datasets, in this study, the challenge of limited data was addressed through the implementation of data augmentation using the multinomial probability distribution and correlations of the items from the local RCADS reports. The intervention of ML models can help in improved accuracy and precision, automated scoring, real-time analysis, longitudinal monitoring, anomaly detection, and contextual analysis. Major contributions of this research are as follow:

- Applied ML to item-level RCADS-47 responses, expanding its application from psychometric validation to predictive psychiatric screening.
- Proposed data augmentation techniques to mitigate the small sample size, to improve the performance and generalizability of models on low resource mental health data.
- Evidenced the appropriateness of RCADS-47 as a holistic and culturally flexible instrument of screening child mental health in Pakistan.

The structure of this document is as follows: The materials and methods employed in this research are described in Section 2, and the results obtained are presented in Section 3. In Section 4, the results are discussed to provide a comprehensive summary of the whole investigation.

## 2 Materials and methods

### 2.1 Participants

The data for the study was provided by the Institute of Psychiatry at Benazir Bhutto Hospital, Rawalpindi which is a teaching hospital that provides basic specialties alongside urology, cardiology, orthopedics, and psychiatry. It is associated with the Medical University of Rawalpindi. The Institute of Psychiatry, the first of its kind in Punjab, holds the distinction of being a regional.

World Health Organization (WHO) center for mental health. Patients granted consent for the use of their RCADS reports, understanding that they would be kept anonymous and the data would be used strictly for research and the Institutional Review Board approved the research proposal (IRB No. 2024-IRB-A-06/06, dated February 22, 2024). The data consisted of RCADS evaluations of 138 children and adolescents, ranging from grade 3 to grade 12.

### 2.2 Internal consistency and reliability analysis

In line with the published studies, each subscale’s internal consistency was measured using Cronbach alpha to see how closely connected the RCADS items were to one another. Cronbach’s alpha (α) quantifies the reliability of a score by calculating the inter-item correlations among all items and the magnitude of Cronbach’s alpha to summarize the information of questionnaire items (39). Alpha values of 0.70 or higher were considered acceptable. The 47 items of the RCADS are divided into 6 subscales, however, since the study focuses on evaluating the internalizing scale, the computation was limited to this scale only. None of the following steps made use of the evaluations from the other subscales.

### 2.3 Feature selection

Feature selection is the step of extracting the most relevant input features before the development of a predictive model to improve the model’s accuracy and efficiency. The study utilized two feature selection techniques to determine which of the 47 independent input variables were the most relevant. The first is the filter method, namely the Chi-square test of independence and Spearman’s correlation, and the second is the wrapper method, namely recursive feature analysis. The chi-square test is one of the most used statistical methods for determining whether two categorical variables are associated or not. The second filter method used is Spearman’s correlation coefficient, named after Charles Spearman, which is a non-parametric measurement that uses ranks to measure the relationship between variables. It measures the degree to which a monotonic function can adequately explain the connection between two variables (40).

The Random Forest-Recursive Feature Elimination (RF-RFE) algorithm was used for the identification of significant features to be used during machine learning as Random Forest, a multiclass algorithm, has an intrinsic unbiased feature significance metric (41). Initially, SVM was used for RFE, however, SVM’s capacity to find strong predictors is impacted by the presence of correlated predictors, even if it supports non-linear connections between predictors. The Random Forest-Recursive Feature Elimination (RF-RFE) method is one proposed solution. Similar applications of the RFE technique to Random Forests have shown that it works well when correlated features are present (42). In this study, a specific number of features to be selected was not provided; instead, the model determined it automatically.

### 2.4 Data augmentation

Machine learning models usually require large quantities of data to ensure satisfactory results. A wide range of regularization strategies are used to enhance model performance, one of which is data augmentation. Data augmentation uses an existing dataset to create data that is computer-generated (43). As stated in Section 3.1, 49 out of the 138 cases were discarded because of missing data, leaving us with 89 instances. 89 instances in a dataset are insufficient to create generalizable machine-learning model. In disciplines like deep learning, progressively expanding the dataset is a common approach that involves artificially increasing the training dataset to improve the performance of the model (44). Additionally, the concept of gradually expanding augmented data is consistent with ensemble methods like bootstrapping and bagging, which provide several data subsets to train various models (45). Therefore, augmented data was generated utilizing the multinomial probability distribution followed by the sex, grade, and 47 items of RCADS. The reason why the multinomial distribution-based augmentation method was chosen is due to its natural distribution to the discrete and ordinal nature of RCADS items that are usually graded on Likert-type scales (0 3). The multinomial method unlike SMOTE does not interpolate the values and therefore maintains the categorical integrity of the values of one item and does not give unrealistic and fractional scores. GANs or VAEs, as generative models, can also model complicated dependencies but would need large sample sizes to be trained with consistency - sometimes impossible in small, sensitive psychiatric datasets. Bootstrapping is easy and simply resamples data, but does not induce new combinations of patterns of symptoms. Multinomial-based sampling, in contrast, samples more data sets with realistically estimated probability bounds that are accurate to the original distribution and are distributionally faithful and interpretable. Therefore, this method offers a statistically clear low-risk augmentation plan that is applicable to small and discrete psychometric data such as RCADS.

The multinomial distribution of the original data was investigated using the chisq.test function of the MASS package in R at an alpha level of 0.05 and the MASS and copula libraries were utilized to generate augmented data that closely replicated the probability distribution of the original data (**Supplementary File 1**). The dataset consists of sex, grade, and 47 items of RCADS. Sex and grade were generated separately using only their probabilities, as correlation analysis showed a poor correlation between these two variables and the 47 features. The RCADS items have four possible outcomes (0 = Never, 1 = Sometimes, 2 = Often, and 3 = Always) which means that it most likely follows a multinomial distribution. To verify this, a chi-square test was conducted on each item, confirming the multinomial distribution (**Supplementary File 2**). This probability distribution was used to generate 5 sets of data (1:4; four times the original data, 1:8; eight times the original data, 1:12; twelve times the original data, 1:16; sixteen times the original data, and 1:20; twenty times the original data) that mimic the distributional properties of the original 89 instances. Given that questions on the same subscale have a moderate correlation with one another, meaning that a child who responds “often” or “always” to one depression-related question is likely to respond similarly to other depression-related questions and similarly for other subscales as well, the data was generated for each subscale. To ensure the synthetic data accurately reflected these patterns and was not made up of random numbers, the average correlation between the questions and the target evaluation of each subscale was calculated and used as input. Additionally, the probability of each possible answer (0, 1, 2, and 3) was also provided as input in the R code. The original 89 and the augmented instances were combined to generate a ‘hybrid’ dataset, which was then used in model development.

In order to test the distributional features between the real and synthetic sets, the subset of 1: 4 ratio was chosen to be analyzed in detail. The Mann Whitney test was used to test each item of the RCADS in order to compare the distributions between the synthetic data and original data. The findings showed that the p-values were all above 0.05, which implies that the distributions of the real and synthetic dataset were not statistically significant. The results have been shown in **Supplementary File 2**, **Supplementary Table S5**. The finding indicates that the synthetic data is effective in maintaining the distributional properties of original data.

### 2.5 Model development and evaluation

Based on RCADS-47 T-scores, cases were classified as normal, borderline and clinical, with 7% in the borderline, 55% in normal and 38% in clinical range after data preprocessing. Synthetic Minority Oversampling Technique (SMOTE) was used to up sample the minority classes in order to tackle the problem of class imbalance in the original data set. Once the data had been balanced, it was split into 80% training and 20% testing data. Six machine learning algorithms were created on the original dataset, the Random Forest (RF), Support Vector Machine (SVM), Logistic Regression (LR), Decision Tree (DT), Naive Bayes (NB), and K-Nearest Neighbors (KNN). The input file for training these models consisted of encoded responses (0 = Never, 1 = Sometimes, 2 = Often, 3 = Always) to the RCADS items, which were treated as features, and the encoded target variable which had three classes (0 = Normal, 1 = Borderline, 2 = Clinical). The feature eliminated during the feature selection step was removed and was not included in the input file. Each model was optimized by means of grid search with five-fold cross-validation (CV = 5). The following parameter grids were used: with RF, the number of estimators (50, 100, 200), the maximum depth (None, 10, 20, 30), the minimum samples split (2, 5, 10), and the minimum samples per leaf (1, 2, 4); with SVM, penalty parameter C (0.1, 1, 10, 100), the type of kernel (linear, rbf), and the value of gamma (‘scale’, auto), with LR, C (regularization) (0.01, 0.1, 1, 10, 100) and penalty (uniform, distance); to DT, maximum depth (None, 10, 20, 30), minimum samples split (2, 5, 10), minimum samples per leaf (1, 2, 4), and criterion (gini, entropy); and to KNN, the number of neighbors (3, 5, 7, 9), the weights (uniform, distance), and the metric (‘euclidean’, ‘manhattan’). The NB model did not have any hyper parameters which were tuned. Cross-validation led to the selection of the best-performing parameters that were used on the test data to evaluate the model. In order to achieve the generalizability, the three best models that had the highest test accuracy were retrained and tested again on an augmented datasets, and the most successful model at this point (based on paired-sample t-test) was again tested on an external dataset. The models’ performance was evaluated using accuracy, precision, recall and F1 score metrics with respect to each class. These metrics track and evaluate the ML algorithms’ performance quality during the training and testing stages and they do so by comparing the classification labels given by the model with the actual labels of the target in the dataset. All the statistical analyses were done using SPSS version 20. RF-RFE and model development was done on Python using the Scikit library and data augmentation was done using R language version 4.4.0 in Rstudio.

## 3 Results

### 3.1 Data pre-processing

Missing data was found for 12 individuals in the ‘Sex’ variable and 46 individuals in the ‘Grades’ variable. Since these two are important variables for RCADS T score evaluation, any case that lacked either of these information was discarded. Instances with even one missing value were deleted, leaving 89 instances, 34 boys (38%) and 55 girls (62%). In order to evaluate the possibility of bias due to missing cases, total RCADS raw scores, were compared between retained (n = 89) and dropped (n = 49) participants. There was no significant difference (t-statistics = -0.039, p-value = 0.968), which means that the cases that were excluded had the same level of symptoms as the research sample and that the missing cases were not likely to create systematic bias. As per RCADS scoring criteria, a total score of less than 65 was categorized as Normal, a score between 65 and 69 as Borderline, and a score of 70 or more as Clinical. In the remaining dataset of 89 individuals, there were 49 normal cases (55%), 6 borderline cases (7%), and 34 clinical cases (38%). Among the 34 boys, 17 were classified as normal (50%), 3 as borderline (9%), and 14 as clinical (41%). **Tables 1**, **2** show the detailed distribution of the instances.

**Table 1:** Distribution of data (gender).

Evaluation				
Gender	Normal	Borderline	Clinical	Total
	n (%)	n (%)	n (%)	
Boys	17 (50%)	3 (9%)	14 (41%)	34 (38%)
Girls	32 (58%)	3 (6%)	20 (36%)	55 (62%)
Total	49 (55%)	6 (7%)	34 (38%)	89

**Table 2:** Distribution of data (grade).

Evaluation				
Grade	Normal	Borderline	Clinical	Total
	n (%)	n (%)	n (%)	
2	1 (100.0)	0 (0.0)	0 (0.0)	1
3	4 (66.6)	1 (16.6)	1 (16.6)	6
4	5 (83.3)	0 (0.0)	1 (16.6)	6
5	2 (100.0)	0 (0.0)	0 (0.0)	2
6	9 (75.0)	0 (0.0)	3 (25.0)	12
7	8 (61.5)	1(7.6)	4 (30.7)	13
8	4 (33.3)	2 (16.6)	6 (50.0)	12
9	6 (37.5)	1 (6.2)	9 (56.2)	16
10	5 (38.4)	1 (7.6)	7 (53.8)	13
11	1 (33.3)	0 (0.0)	2 (66.6)	3
12	4 (80.0)	0 (0.0)	1 (20.0)	5
Total	49 (55)	6(7)	34 (38)	89

### 3.2 Internal consistency and reliability analysis

The internal consistency of RCADS’ overall internalizing scale, overall anxiety scale, and each subscale was assessed using Cronbach’s alpha. RCADS showed excellent internal consistency with an alpha of 0.953 (**Table 3**). The average inter-item correlation for the 47 items was found to be weak to moderate, with the majority of correlations lying between 0.1 and 0.6. Removing items 3 and 5 resulted in a slight increase in the scale’s internal consistency from 0.953 to 0.954. Conversely, removing items 10, 12, 15, 18, 19, 20, 21, 22, 23, 25, 29, 30, 31, 32, 34, 35, 37, 38, 39, 40, 41, 42, 44, and 45 caused a decrease from 0.953 to 0.952. Additionally, the removal of items 27 and 47 lowered the consistency from 0.953 to 0.951. However, these changes are too minor to be considered significant. Within each subscale, removing any item from the Major Depressive Disorder subscale, Obsessive-Compulsive Disorder subscale, Separation Anxiety Disorder subscale, and Social Phobia subscale reduced their internal consistency. For the Generalized Anxiety Disorder subscale, the removal of item 13 increased its internal consistency from 0.753 to 0.768. The subscale’s internal consistency decreased when any other item was deleted. When item 3 from the Panic Disorder subscale was eliminated, the internal consistency of the subscale improved slightly from 0.810 to 0.817.

**Table 3:** Internal consistency coefficient Cronbach’s alpha for each subscale.

Subscale	Cronbach’s alpha
Overall Internalizing Scale	0.953
Overall Anxiety Scale	0.940
Major Depressive Disorder (MDD)	0.859
Generalized Anxiety Disorder (GAD)	0.753
Obsessive-Compulsive Disorder (OCD)	0.747
Panic Disorder (PD)	0.810
Separation Anxiety Disorder (SAD)	0.761
Social Phobia (SP)	0.835

### 3.3 Feature selection

Both filter and wrapper methods analysis revealed that the majority of the features demonstrated a significant correlation with the target variable and played a crucial role in the final evaluation. The chi-square test of independence revealed that most of the features were statistically significant at an alpha level of 0.05. Similarly, the correlation analysis between the target variable and the features showed that most features had a significant correlation. At the 0.05 alpha level, item 05 did not show a significant correlation with the target variable. However, it is important to highlight that all correlations were significant at 0.01 alpha level. RF-RFE selected 35 features as important to train a model with an accuracy of 88%. The number of features eliminated by the Chi-square test of independence, Spearman’s correlation, and RF-RFE are four, one, and twelve respectively (**Supplementary File 2**). Rcads05 was consistently identified as insignificant by all three methods. Therefore, it was eliminated from the data and not used during model training.

### 3.4 Model development and evaluation

The Synthetic Minority Oversampling Technique (SMOTE) was used to over sample the minority classes to deal with the issue of class imbalance. The dataset was then split in training (80%) and testing (20%) datasets, which made the shapes of the data. *X*_train = (117, 46), *X*_test = (30, 46), *y*_train = (117), and *y*_test = (30),. There were six machine learning algorithms, which are the Random Forest (RF), Support Vector Machine (SVM), Logistic Regression (LR), Decision Tree (DT), Naive Bayes (NB), and K-Nearest Neighbors (KNN), which were generated and tuned with grid search on the training set with a cross-validation of five-fold cross-validation (CV = 5) to identify the best hyper parameter settings of each algorithm. The best parameters and the associated average cross-validation accuracies were the following:

- Random Forest (RF): n_estimators=50, max depth= none, min samples split=5, min samples leaf=2, and obtaining the cross-validation accuracy of 0.96.
- Support Vector Machine (SVM): C = 1, gamma = scale and kernel = rbf and the cross-validation accuracy of 0.97 is the highest.
- Logistic Regression (LR): C = 100 and penalty = l1 and the accuracy is 0.90.
- Decision Tree (DT): criterion = gini, max-depth = none, min-samples- split = 2 and min-samples-leaf = 2 with the resultant accuracy of 0.82.
- Naive Bayes (NB): average cross-validation accuracy of 0.92.
- K-Nearest Neighbors (KNN): n neighbors = 3, weights = distance, and metric = Manhattan, which had a result of 0.92.

Once the best parameters were chosen, all the 6 models with best parameters were tested on the test dataset in order to predict their performance. Random Forest model had a general test accuracy of 0.87 and F1-scores of 0.80, 0.93, and 0.88 between the normal, borderline and clinical classes respectively. The Support Vector Machine (SVM) model had the highest test accuracy of 0.97 with a macro average precision, recall and F1-scores of 0.97 for each, showing high levels of classification across all the classes. Logistic Regression model generated a test accuracy of 0.83, which was moderate in terms of precision, and recall, especially the normal class. The Decision Tree model also obtained the accuracy of 0.83 and balanced performance across the classes (macro average F1 = 0.84). Naive Bayes model has achieved a precision of 0.87 with the borderline and clinical classes having high recall and somewhat low precision with the normal class. The best model was the K-Nearest Neighbors that registered a test-based accuracy of 0.90 with macro average precision, recall and F1-scores of 0.90, 0.91 and 0.89 respectively. Detailed results are shown in **Table 4**. SVM, KNN and RF are among the considered models, which performed better in general and were also more stable in both the training and the testing stages. The three best models thus were chosen to be further evaluated on the generalizability by the augmented dataset.

**Table 4:** Results of machine learning models on the test set of original data concerning their post-SMOTE application and hyper parameter optimization.

Class	Precision	Recall	F1-score	Model
0	0.89	0.73	0.8	Random Forest
1	0.88	1	0.93	
2	0.85	0.92	0.88	
accuracy			0.87	
macro average	0.87	0.88	0.87	
weighted average	0.87	0.87	0.86	
0	1	0.91	0.95	SVM
1	1	1	1	
2	0.92	1	0.96	
accuracy			0.97	
macro average	0.97	0.97	0.97	
weighted average	0.97	0.97	0.97	
0	0.86	0.64	0.74	Logistic Regression
1	0.78	1	0.88	
2	0.85	0.92	0.88	
accuracy			0.83	
macro average	0.84	0.85	0.83	
weighted average	0.84	0.83	0.83	
0	0.75	0.82	0.78	Decision Tree
1	0.86	0.86	0.86	
2	0.91	0.83	0.87	
accuracy			0.83	
macro average	0.84	0.84	0.84	
weighted average	0.84	0.83	0.83	
0	1	0.64	0.78	Naive Bayes
1	0.78	1	0.88	
2	0.86	1	0.92	
accuracy			0.87	
macro average	0.88	0.88	0.86	
weighted average	0.89	0.87	0.86	
0	1	0.73	0.84	KNN
1	0.78	1	0.88	
2	0.92	1	0.96	
accuracy			0.9	
macro average	0.90	0.91	0.89	
weighted average	0.92	0.9	0.90	

In order to measure the model robustness and generalizability, it was first assembled on five augmented datasets with augmentation ratios of 1:4, 1:8, 1:12, 1:16, and 1:20. Three top models of the last step, namely, Random Forest (RF), Support Vector Machine (SVM), and K-Nearest Neighbors (KNN) were trained and tested on every augmented dataset with five-fold cross-validation. In all augmented datasets, RF cross-validation mean values were between 0.81 and 0.82, SVM between 0.79 and 0.83 and KNN between 0.77 and 0.81. The best mean accuracies were with the 1:4 augmented data where RF, SVM and KNN had 0.82, 0.83 and 0.81 respectively. These findings suggest that moderate augmentation (1:4) was better at generalization than smaller datasets, while larger augmentation ratios did not yield further performance gains. Though the average performances of all the models on augmented datasets were slightly lower than that they obtained on the original dataset, this decrease is an indication of a trend toward improved model generalization as opposed to over-fitting to the original data distribution. A moderate augmentation (1:4) added enough variability to enhance robustness without compensating the representativeness of the actual data. On the contrary, the performance gains leveled off and in certain instances decreased with an augmentation ratio exceeding 1:4. It is possible to explain this plateauing effect through the effect of synthetic redundancy, that is, the production of synthetic samples which are too similar to the data at hand, and which do not add a lot of information to it. Over-augmentation is likely to decrease the variety of data and make the model repeat the same redundant patterns instead of acquiring novel signal variations. This not only precludes any further gain in accuracy but can also blur significant differences between classes. In turn, the 1:4 augmentation ratio was considered the most suitable because it did not compromise the information but also expanded the learning feature space.

Paired t-tests were used to statistically compare the model performance of the augmented datasets using the 5 fold cross-validation results. In the case of the 1:4 set, there were no significant differences in the results of SVM, RF, and KNN (SVM vs. RF: p-value = 0.76; SVM vs. KNN: p-value = 0.32; RF vs. KNN: p-value = 0.46) indicating there were no major differences in the predictive abilities of the models. Nevertheless, both in terms of quantitative performance and theoretical justification, the Random Forest model was chosen as a final model that would be further externally validated. Besides attaining the competitive cross-validation accuracy on augmented datasets, RF also showed better performance in the test-set on the 1: 4 augmented data with the accuracy of 81%, as compared to both SVM and KNN, which had an accuracy of 77%, as indicated in **Table 5**. In addition, RF is much more interpretable than SVM and KNN due to its ranking of feature importance that can be especially useful in the analysis of psychological or behavioral data. This interpretability, together with the fact that it is stable across various augmented datasets and equally balanced in terms of generalization, justifies the choice of RF as the most suitable and reliable model to be used in this study.

**Table 5:** Test set performance of top 3 models across augmented dataset (1:4).

Class	Precision	Recall	F1-score	Model
0	0.83	0.75	0.78	Random Forest
1	0.81	0.83	0.82	
2	0.80	0.87	0.83	
Accuracy			0.81	
0	0.79	0.67	0.72	SVM
1	0.68	0.83	0.75	
2	0.86	0.82	0.84	
Accuracy			0.77	
0	0.90	0.51	0.65	KNN
1	0.68	0.99	0.8	
2	0.85	0.87	0.86	
Accuracy			0.77	

**Figure 1:**
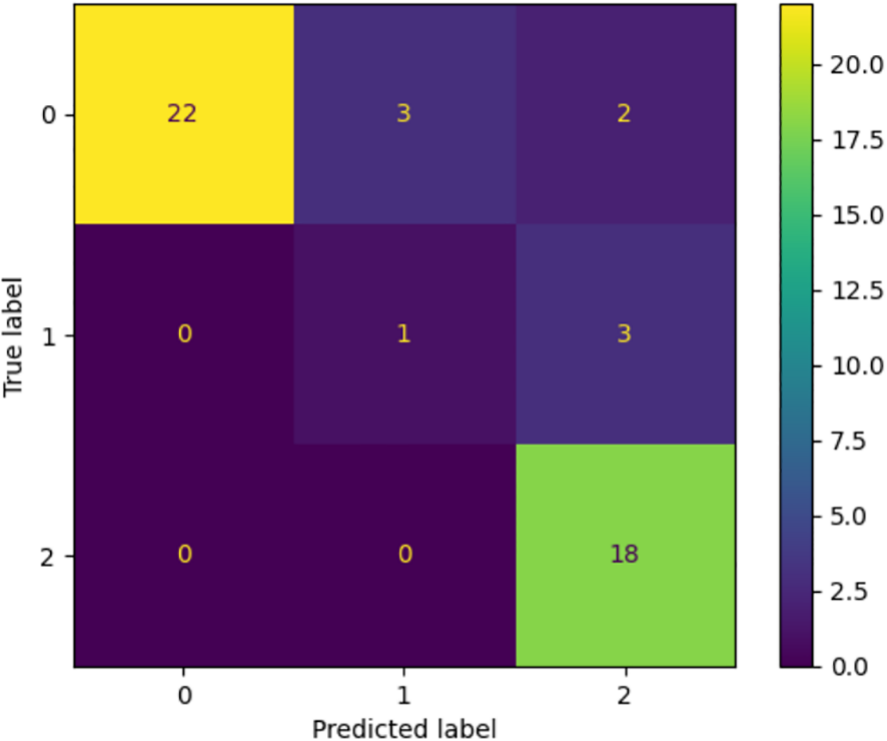
Confusion matrix with the performance of the Random Forest model on the external dataset. The model is very strong in the classification of normal and clinical classes and low in the classification of the borderline one, with many false classifications in the nearest categories. The trend can be attributed to the fact that there is a natural overlap of the class lines and a lack of representation of the borderline cases in training.

In order to further test the generalizability of Random Forest (RF), external validation on a different dataset was conducted. **Figure 1** contains the corresponding confusion matrix that has shown the model outcomes in classification with regard to the three categories, namely, normal, borderline and clinical. The RF model showed great predictive accuracy when compared with the normal and clinical group and accurately predicted most of the cases in the respective classes. Nevertheless, the borderline class experienced higher degree of misclassification with a significant percentage of the samples being predicted as clinical. It is therefore evident that though the model has a high rate of accurately discriminating definite cases, its ability to detect weak or concrete cases is limited. The given pattern suggests the overlap of the conceptualization of the classes on the RCADS scale, as well as the relatively low proportion of borderline cases in the training data, which together limit the applicability of the model across the datasets.

## 4 Discussion

The onset of nearly half of all mental illnesses occurs around the age of 14, and unspecified mental and social disturbances are often the precursor to major mental disorders. These disturbances can progress into any major mental disorder and account for 45% of the worldwide disease burden among individuals aged 0 to 25 (46). Therefore, identifying mental health problems at their earliest stages is crucial. This is particularly important in Pakistan, where approximately 15 million individuals face mental health challenges, and with more than half of the population under 18, a significant portion of those affected are likely to be children and adolescents. Studies have shown that there is a treatment gap for mental illness in low- and middle-income countries (LMICs), where over 90% of patients do not access affordable therapies (47). The shortage and uneven distribution of mental health professionals are major barriers to closing this gap (47–49). Because of this treatment gap and related workforce challenges, there is a need to maximize the utilization of currently available healthcare resources. This can be achieved by designing effective depression and anxiety screening models that can be used by healthcare professionals with less experience and training as well.

In this study, we proposed an ML-based screening method using RCADS-47 for the early identification of depression and anxiety. The Revised Child Anxiety and Depression Scale (RCADS) has established itself as a widely utilized self-report tool for diagnosing anxiety and depression symptoms in children and adolescents but the majority of the RCADS validation has been carried out in Western countries. Evaluating it in developing countries, where anxiety and depression prevalence differs, is also necessary. Therefore, the internal consistency of RCADS was calculated. RCADS demonstrated weak to moderate inter-item correlations, indicating that while the questionnaire’s items are focused on one particular disorder, they are well-diversified to avoid being redundant or repetitive. The scale also showed strong internal consistency for all the subscales, suggesting their significance for the scale’s administration. These findings are consistent with published research on the tool’s internal consistency. Additionally, we explored data augmentation to tackle the problem of small data size which proved to be a viable substitute for real-world data. Applications of the synthetic data in this research would help to manage critical ethical issues that come with psychiatric studies in low- and middle-income countries (LMICs), including Pakistan. Mental illness stigmatization makes it hard to underreport and participate in investigations, which restrains access to the data. We reduce the privacy risks by creating synthetic data with the statistical characteristics of actual responses, but without the individual identities, allowing ethical data sharing to develop models. This will be an inclusive method of research on mental health and will uphold the privacy of the research participants. However, data generation and validation need to be transparent to prevent biases or misunderstandings, where synthetic data should be used to complement, not to substitute real-life evidences in the culturally sensitive psychiatric research.

Feature selection methods revealed that item 05 (“I would feel afraid of being on my own at home”) did not have a significant correlation with the evaluation of depression and anxiety in the study population. The elimination of this question is understandable as in Pakistani culture joint families are common, and children are rarely left at home alone. While teenage boys may have some unsupervised time, it is less common for younger children and teenage girls. As a result, the feeling of being afraid when alone at home is not a typical experience for most children in Pakistan. This might explain why the particular question concerning this fear had no meaningful impact on evaluating feelings of depression or anxiety. The developed machine learning models achieved good classification accuracy and F1 scores with Random Forest achieving the highest F1 score. In multi-class classification, choosing the optimal model based on the F1 score is helpful as it guarantees a balance between recall and precision, offering a thorough assessment of model performance (50). It makes model comparison easier by providing a single statistic that takes into account both false positives and false negatives. As it may be weighted, macro, or averaged to represent performance across classes, it is well suited for multi-class settings and ensures consistency (51). Out of the three target classes, the ‘borderline’ class was frequently observed to be falsely classified as the ‘normal’ class. The lack of borderline cases in the original data likely contributed to the poor representation of this class since it is difficult for machine learning models to identify the underlying patterns in minority classes, which results in incorrect categorization. Random Forest had the best overall performance compared to the other algorithms on synthetic data. Moreover, Random Forest is well-suited for categorical data, which aligns perfectly with RCADS questionnaire responses that are categorical (0 = never, 1 = sometimes, 2 = often, 3 = always) and it provides probability estimates for each class, making it easier to handle uncertainty and ambiguity in responses. This is particularly useful in psychological assessments where responses can be subjective (52). Therefore, this algorithm seems to be an effective decision support system to help medical practitioners make well-informed screening decisions based on the chosen RCADS features. Notably, a primary aim of the research accomplished by earlier researchers on RCADS in the past has been on the validation, reliability, and cross-cultural adaptation, but no studies have yet applied machine learning techniques to model or predict the results of the RCADS. Our work is thus innovative in its combination of data augmentation and ML classification with intuitive findings that models like Random Forest are useful in representing underlying symptom structures predictive of known psychometric patterns, and in extending the applications of RCADS to data-driven screening in low-resource and high-stigma settings.

In developing countries, where anxiety and depression prevalence differs and where research is scarce and the burden of poor mental health is made worse by several issues like societal stigma, limited access to resources, and the high cost of mental health consultations, this preliminary contribution to the field of mental health can encourage more research and development concerning the integration of ML in healthcare practices. Furthermore, we recommend implementing this approach within the education system. Since schools are where children spend a large portion of their time, they are the most suitable places to implement comprehensive mental health services. Additionally, research from high-income countries (HICs) shows there is a strong correlation between educational failure and childhood mental disorders (53, 54), underscoring the need for mental health services in the educational setting. Academic progress and general well-being can be enhanced by establishing a continuum of treatment through the integration of mental health screening into the school setting given that school-based interventions (SBIs) have been proven to be effective treatments for improving child mental health (55).

### 4.1 Strengths and limitations

There are certain limitations associated with this study. First, the size of the dataset was relatively small and this can restrict the generalizability of the machine learning models even after cross-validation. To reduce this we used data augmentation but doing so brings about some risks, such as subtle distributional biases and the fact that an augmented sample may not fully represent the complexity of real world responses. Even though statistical tests proved that there was no significant difference in the distributional variations between real and synthetic data, the results are to be treated carefully. Future studies should confirm these findings with more extensive multi-site data and examine the various strategies of augmentation in order to enhance reliability and guarantee strong generalization with very diverse populations.

## 5 Conclusion

This study is the first to use machine learning techniques on RCADS-47 data, extending its use from psychometric validation, to predictive modeling in child mental health. The combination of multinomial distribution-based data augmentation with ML classification helped to overcome the problem of small, psychiatric data that is common in low- and middle-income countries. Not only did the method maintain the statistical characteristics of the original data, but also improved the generalizability of model to show that synthetic data can be effectively and ethically utilized to conduct research in mental health. The most robust and interpretable results were obtained with the Random Forest model that implies its applicability in screening applications. The results of the current research can be potentially useful as a screening support tool in both schools and outpatient hospitals in Pakistan and other LMIC countries. Instead of being a diagnostic tool, the model can assist clinicians, psychologists, and school counselors to identify the students that might be subjected to additional testing. Nevertheless, one should pay closer attention to the possibility of a false negative since the latent cases might cause a delay in timely intervention. To reduce this risk, the tool must be employed as one of the levels of screening process, along with clinical interview and teacher or parent report. Such an ML-based system when introduced within current mental health models would involve improvements in the initial diagnosis without violating ethical standards and clinical supervision. In general, this paper adds a new framework integrating psychometric rigor, data augmentation, and machine learning to expand culturally flexible and privacy-preserving psychiatric screening instruments in resource-constrained settings.

## Data Availability

The raw data supporting the conclusions of this article will be made available by the authors, without undue reservation.
