# Supplementary material for: Leveraging data augmentation for machine learning models in predicting depression and anxiety using the Revised Child Anxiety and Depression Scale clinical reports
Source: Front Psychiatry. 2025 Nov 27;16:1672178. doi: 10.3389/fpsyt.2025.1672178 (PMC12696576; doi:10.3389/fpsyt.2025.1672178)
Supplement: Supplementary file 1 [file Table1.docx]

**Supplementary Codes**

**Python code for RF-RFE**

# import packages

from google.colab import files

import pandas as pd

# Uploading the CSV file from computer

uploaded = files.upload()

filename = 'data.csv'

df = pd.read_csv(filename)

print(df)

# Separate the features and target variables

X = df.drop('Target', axis=1)

y = df['Target']

import numpy as np

import matplotlib.pyplot as plt

from sklearn.datasets import make_classification

from sklearn.model_selection import cross_val_score, StratifiedKFold

from sklearn.feature_selection import RFE

from sklearn.tree import DecisionTreeClassifier

from sklearn.ensemble import RandomForestClassifier

from sklearn.pipeline import Pipeline

# Function to evaluate a model using stratified 5-fold cross-validation

def evaluate_model(model, X, y):

cv = StratifiedKFold(n_splits=5, shuffle=True, random_state=42)

scores = cross_val_score(model, X, y, scoring='accuracy', cv=cv, n_jobs=-1)

return np.mean(scores)

# Initialize a RandomForestClassifier as the estimator

estimator = RandomForestClassifier(n_estimators=100, random_state=42)

# Initialize a list to store the mean accuracies for each number of selected features

mean_accuracies = []

# Initialize a list to store the names of the optimal number of selected features

optimal_feature_counts = []

# Loop through different numbers of selected features

for i in range(1, 48):

rfe = RFE(estimator=estimator, n_features_to_select=i)

model = RandomForestClassifier()

pipeline = Pipeline(steps=[('s', rfe), ('m', model)])

mean_accuracy = evaluate_model(pipeline, X, y)

mean_accuracies.append(mean_accuracy)

optimal_feature_counts.append(i)

print(f"Number of Selected Features: {i}, Mean Accuracy: {mean_accuracy:.3f}")

# Find the index of the maximum mean accuracy

optimal_idx = np.argmax(mean_accuracies)

optimal_features = optimal_feature_counts[optimal_idx]

print(f"Optimal Number of Selected Features: {optimal_features}")

# Plot feature importances using the RandomForestClassifier

estimator.fit(X, y)

importances = estimator.feature_importances_

print(importances)

plt.figure(figsize=(20, 16))

plt.title("Feature Importances")

plt.bar(range(len(importances)), importances, tick_label=np.arange(1, len(importances) + 1))

plt.xlabel("Feature Number")

plt.ylabel("Importance")

plt.show()

**R code for the chi-square test for multinomial distribution**

# Load libraries

library(MASS) # for chisq.test

# Read data

data=read.csv("RCADS.csv",header=TRUE)

# Check for missing values (not recommended for multinomial)

if (any(is.na(data$rcads))) {

stop("Data contains missing values. Multinomial not suitable!")

}

# Get observed counts

observed <- table(data$rcads)

# Define total number of trials (observations)

n <- sum(observed)

# Define the vector of unequal probabilities (replace with your values)

p <- c(0.08, 0.07, 0.02, 0.14, 0.13, 0.14, 0.18, 0.15, 0.03, 0.06) # Probabilities for categories 0, 1, 2, 3

# Check if probability vector length matches category count

if (length(p) != nrow(table(data$rcads))) {

stop("Probability vector length must match number of categories!")

}

# Calculate expected counts using the probabilities

expected <- n * p

# Create table for observed and expected counts

counts_table <- cbind(Category = names(observed), Observed = observed, Expected = expected)

# Print the table

cat("Table of Observed and Expected Counts:\n")

print(counts_table)

# Perform Chi-squared goodness-of-fit test

chisq.result <- chisq.test(observed, p = expected/n)

# Print results

cat("Chi-squared test for multinomial distribution with unequal probabilities:\n")

print(chisq.result)

# Interpret results

if (chisq.result$p.value > 0.05) {

cat("p-value =", chisq.result$p.value,

"\nWe fail to reject the null hypothesis of multinomial fit.\n")

} else {

cat("p-value =", chisq.result$p.value,

"\nWe reject the null hypothesis of multinomial fit.\n")

}

**R code for data augmentation**

# Load libraries

library(copula)

library(MASS)

# Define the correlation

correlation <- 0.35

# Number of samples

n <- 348

# Number of series

num_series <- 6

# Generate a multivariate normal distribution with the specified correlation matrix

sigma <- matrix(correlation, num_series, num_series) + diag(1 - correlation, num_series)

normals <- mvrnorm(n, mu = rep(0, num_series), Sigma = sigma)

# Transform the normal variables to a uniform using the CDF

uniforms <- pnorm(normals)

# Define the probabilities for the multinomial distributions

probs_list <- list(

c(0.07, 0.43, 0.23, 0.27),

c(0.39, 0.30, 0.15, 0.16),

c(0.30, 0.26, 0.23, 0.21),

c(0.38, 0.29, 0.20, 0.13),

c(0.22, 0.35, 0.24, 0.19),

c(0.36, 0.26, 0.16, 0.22))

# Ensure the number of probability vectors matches the number of series

if (length(probs_list) != num_series) {

stop("The length of probs_list must match the number of series.")

}

# Function to map uniform variables to multinomial

uniform_to_multinomial <- function(u, probs) {

return(findInterval(u, cumsum(probs), rightmost.closed = TRUE))

}

# Generate the series of multinomial random numbers

series_list <- lapply(1:num_series, function(i) {

sapply(uniforms[, i], uniform_to_multinomial, probs = probs_list[[i]])

})

# Combine the series into a data frame

result <- as.data.frame(do.call(cbind, series_list))

colnames(result) <- paste0("series", 1:num_series)

# Save to a CSV file

write.csv(result, file = "GAD_synthetic.csv", row.names = TRUE)

# Verify the correlation

print(cor(result))
