# Supplementary material for: Leveraging data augmentation for machine learning models in predicting depression and anxiety using the Revised Child Anxiety and Depression Scale clinical reports
Source: Front Psychiatry. 2025 Nov 27;16:1672178. doi: 10.3389/fpsyt.2025.1672178 (PMC12696576; doi:10.3389/fpsyt.2025.1672178)
Supplement: Supplementary file 2 [file Table2.docx]

**Supplementary Tables**

Supplementary Table S1. Result of chi-square test of independence of the 47 features with the target

| **Feature** | **χ2** | **C** | **p-value** |
| --- | --- | --- | --- |
| Rcads01 | 23.872 | 0.464 | 0.001 |
| Rcads02 | 14.159 | 0.374 | 0.028 |
| Rcads03 | 13.018 | 0.361 | 0.043 |
| Rcads04 | 16.275 | 0.397 | 0.012 |
| Rcads05 | 4.510 | 0.222 | 0.608 |
| Rcads06 | 17.106 | 0.405 | 0.009 |
| Rcads07 | 19.475 | 0.428 | 0.003 |
| Rcads08 | 11.214 | 0.338 | 0.082 |
| Rcads09 | 10.092 | 0.322 | 0.121 |
| Rcads10 | 22.137 | 0.450 | 0.001 |
| Rcads11 | 16.500 | 0.399 | 0.011 |
| Rcads12 | 29.832 | 0.505 | 0.000 |
| Rcads13 | 24.594 | 0.469 | 0.000 |
| Rcads14 | 22.982 | 0.457 | 0.001 |
| Rcads15 | 40.828 | 0.565 | 0.000 |
| Rcads16 | 18.602 | 0.420 | 0.005 |
| Rcads17 | 8.003 | 0.290 | 0.238 |
| Rcads18 | 33.012 | 0.524 | 0.000 |
| Rcads19 | 22.841 | 0.456 | 0.001 |
| Rcads20 | 28.794 | 0.499 | 0.000 |
| Rcads21 | 39.110 | 0.557 | 0.000 |
| Rcads22 | 34.0.29 | 0.530 | 0.000 |
| Rcads23 | 41.744 | 0.569 | 0.000 |
| Rcads24 | 29.194 | 0.501 | 0.000 |
| Rcads25 | 29.994 | 0.506 | 0.000 |
| Rcads26 | 23.344 | 0.460 | 0.001 |
| Rcads27 | 48.0.95 | 0.597 | 0.000 |
| Rcads28 | 18.858 | 0.422 | 0.004 |
| Rcads29 | 29.697 | 0.504 | 0.000 |
| Rcads30 | 31.671 | 0.517 | 0.000 |
| Rcads31 | 18.673 | 0.420 | 0.005 |
| Rcads32 | 46.902 | 0.592 | 0.000 |
| Rcads33 | 15.315 | 0.387 | 0.018 |
| Rcads34 | 42.000 | 0.571 | 0.000 |
| Rcads35 | 34.964 | 0.535 | 0.000 |
| Rcads36 | 14.343 | 0.376 | 0.026 |
| Rcads37 | 34.975 | 0.535 | 0.000 |
| Rcads38 | 33.155 | 0.525 | 0.000 |
| Rcads39 | 25.125 | 0.473 | 0.000 |
| Rcads40 | 40.651 | 0.564 | 0.000 |
| Rcads41 | 38.008 | 0.551 | 0.000 |
| Rcads42 | 21.330 | 0.444 | 0.002 |
| Rcads43 | 24.121 | 0.466 | 0.000 |
| Rcads44 | 29.094 | 0.501 | 0.000 |
| Rcads45 | 37.894 | 0.551 | 0.000 |
| Rcads46 | 14.511 | 0.378 | 0.024 |
| Rcads47 | 53.447 | 0.617 | 0.000 |
| Features indicating weak associations are highlighted in yellow.  χ²: Chi-square test statistic, C: Contingency Coefficient of Chi-square test. | | | |

Supplementary Table S2. Result of Spearman correlation of the 47 features with the target

| **Feature** | **ρ** | **p-value** |
| --- | --- | --- |
| Rcads01 | 0.461 | 0.000 |
| Rcads02 | 0.271 | 0.011 |
| Rcads03 | 0.241 | 0.025 |
| Rcads04 | 0.353 | 0.001 |
| Rcads05 | 0.158 | 0.143 |
| Rcads06 | 0.351 | 0.001 |
| Rcads07 | 0.449 | 0.000 |
| Rcads08 | 0.267 | 0.013 |
| Rcads09 | 0.309 | 0.004 |
| Rcads10 | 0.470 | 0.000 |
| Rcads11 | 0.410 | 0.000 |
| Rcads12 | 0.567 | 0.000 |
| Rcads13 | 0.471 | 0.000 |
| Rcads14 | 0.426 | 0.000 |
| Rcads15 | 0.552 | 0.000 |
| Rcads16 | 0.434 | 0.000 |
| Rcads17 | 0.240 | 0.025 |
| Rcads18 | 0.601 | 0.000 |
| Rcads19 | 0.472 | 0.000 |
| Rcads20 | 0.500 | 0.000 |
| Rcads21 | 0.621 | 0.000 |
| Rcads22 | 0.603 | 0.000 |
| Rcads23 | 0.623 | 0.000 |
| Rcads24 | 0.541 | 0.000 |
| Rcads25 | 0.519 | 0.000 |
| Rcads26 | 0.476 | 0.000 |
| Rcads27 | 0.666 | 0.000 |
| Rcads28 | 0.357 | 0.001 |
| Rcads29 | 0.525 | 0.000 |
| Rcads30 | 0.564 | 0.000 |
| Rcads31 | 0.442 | 0.000 |
| Rcads32 | 0.684 | 0.000 |
| Rcads33 | 0.376 | 0.000 |
| Rcads34 | 0.626 | 0.000 |
| Rcads35 | 0.568 | 0.000 |
| Rcads36 | 0.374 | 0.000 |
| Rcads37 | 0.555 | 0.000 |
| Rcads38 | 0.576 | 0.000 |
| Rcads39 | 0.516 | 0.000 |
| Rcads40 | 0.646 | 0.000 |
| Rcads41 | 0.601 | 0.000 |
| Rcads42 | 0.420 | 0.000 |
| Rcads43 | 0.458 | 0.000 |
| Rcads44 | 0.563 | 0.000 |
| Rcads45 | 0.633 | 0.000 |
| Rcads46 | 0.327 | 0.002 |
| Rcads47 | 0.727 | 0.000 |
| The feature indicating weak correlation is highlighted in yellow.  ρ: Spearman Correlation Coefficient | | |

Supplementary Table S3. Result of recursive feature elimination – Random Forest

| **Feature** | **Importance calculated by RF-RFE** |
| --- | --- |
| Rcads01 | 0.01934744 |
| Rcads02 | 0.00801127 |
| Rcads03 | 0.00630323 |
| Rcads04 | 0.01530331 |
| Rcads05 | 0.00539821 |
| Rcads06 | 0.00792677 |
| Rcads07 | 0.01147243 |
| Rcads08 | 0.01206183 |
| Rcads09 | 0.00876527 |
| Rcads10 | 0.0082231 |
| Rcads11 | 0.01511179 |
| Rcads12 | 0.01863023 |
| Rcads13 | 0.01440013 |
| Rcads14 | 0.01742943 |
| Rcads15 | 0.04112295 |
| Rcads16 | 0.01306844 |
| Rcads17 | 0.00931252 |
| Rcads18 | 0.02430123 |
| Rcads19 | 0.00848042 |
| Rcads20 | 0.01602037 |
| Rcads21 | 0.04199492 |
| Rcads22 | 0.01713688 |
| Rcads23 | 0.03062126 |
| Rcads24 | 0.02536264 |
| Rcads25 | 0.01492744 |
| Rcads26 | 0.01447971 |
| Rcads27 | 0.03599548 |
| Rcads28 | 0.00813265 |
| Rcads29 | 0.01858358 |
| Rcads30 | 0.03970481 |
| Rcads31 | 0.01797364 |
| Rcads32 | 0.07486209 |
| Rcads33 | 0.00338783 |
| Rcads34 | 0.04569646 |
| Rcads35 | 0.01150855 |
| Rcads36 | 0.00800992 |
| Rcads37 | 0.03303595 |
| Rcads38 | 0.03302176 |
| Rcads39 | 0.01136269 |
| Rcads40 | 0.03999579 |
| Rcads41 | 0.03630199 |
| Rcads42 | 0.01651201 |
| Rcads43 | 0.01410981 |
| Rcads44 | 0.03115129 |
| Rcads45 | 0.02592093 |
| Rcads46 | 0.00605962 |
| Rcads47 | 0.0634599 |
| The features with the low importance score are highlighted in yellow. | |

Supplementary Table S4. Results of the chi-square test for multinomial distribution

| **RCADS Item** | **Chi-square** | **df** | **p-value** | **Null Hypothesis** |
| --- | --- | --- | --- | --- |
| Rcads01 | 0.0169 | 3 | 0.9994 | Fail to reject |
| Rcads02 | 0.04023 | 3 | 0.9979 | Fail to reject |
| Rcads03 | 0.036824 | 3 | 0.9981 | Fail to reject |
| Rcads04 | 0.0275 | 3 | 0.9988 | Fail to reject |
| Rcads05 | 0.016113 | 3 | 0.9995 | Fail to reject |
| Rcads06 | 0.016021 | 3 | 0.9995 | Fail to reject |
| Rcads07 | 0.012091 | 3 | 0.9996 | Fail to reject |
| Rcads08 | 0.0054788 | 3 | 0.9999 | Fail to reject |
| Rcads09 | 0.022784 | 3 | 0.9991 | Fail to reject |
| Rcads10 | 0.03202 | 3 | 0.9985 | Fail to reject |
| Rcads11 | 0.011431 | 3 | 0.9997 | Fail to reject |
| Rcads12 | 0.0051168 | 3 | 0.9999 | Fail to reject |
| Rcads13 | 0.0011789 | 3 | 0.9999 | Fail to reject |
| Rcads14 | 0.0091817 | 3 | 0.9998 | Fail to reject |
| Rcads15 | 0.023593 | 3 | 0.9990 | Fail to reject |
| Rcads16 | 0.015973 | 3 | 0.9995 | Fail to reject |
| Rcads17 | 0.017718 | 3 | 0.9994 | Fail to reject |
| Rcads18 | 0.016254 | 3 | 0.9995 | Fail to reject |
| Rcads19 | 0.0054946 | 3 | 0.9999 | Fail to reject |
| Rcads20 | 0.0082746 | 3 | 0.9998 | Fail to reject |
| Rcads21 | 0.01792 | 3 | 0.9994 | Fail to reject |
| Rcads22 | 0.010762 | 3 | 0.9997 | Fail to reject |
| Rcads23 | 0.0069459 | 3 | 0.9998 | Fail to reject |
| Rcads24 | 0.0098667 | 3 | 0.9997 | Fail to reject |
| Rcads25 | 0.0098667 | 3 | 0.9997 | Fail to reject |
| Rcads26 | 0.0060208 | 3 | 0.9999 | Fail to reject |
| Rcads27 | 0.031392 | 3 | 0.9985 | Fail to reject |
| Rcads28 | 0.030651 | 3 | 0.9986 | Fail to reject |
| Rcads29 | 0.0091817 | 3 | 0.9998 | Fail to reject |
| Rcads30 | 0.010762 | 3 | 0.9997 | Fail to reject |
| Rcads31 | 0.010914 | 3 | 0.9997 | Fail to reject |
| Rcads32 | 0.0522 | 3 | 0.9969 | Fail to reject |
| Rcads33 | 0.000346 | 3 | 0.9999 | Fail to reject |
| Rcads34 | 0.018751 | 3 | 0.9993 | Fail to reject |
| Rcads35 | 0.021728 | 3 | 0.9992 | Fail to reject |
| Rcads36 | 0.011063 | 3 | 0.9997 | Fail to reject |
| Rcads37 | 0.011137 | 3 | 0.9997 | Fail to reject |
| Rcads38 | 0.15112 | 3 | 0.9851 | Fail to reject |
| Rcads39 | 0.014231 | 3 | 0.9996 | Fail to reject |
| Rcads40 | 0.0098101 | 3 | 0.9997 | Fail to reject |
| Rcads41 | 0.010177 | 3 | 0.9997 | Fail to reject |
| Rcads42 | 0.037352 | 3 | 0.9981 | Fail to reject |
| Rcads43 | 0.11431 | 3 | 0.9997 | Fail to reject |
| Rcads44 | 0.010983 | 3 | 0.9997 | Fail to reject |
| Rcads45 | 0.000579 | 3 | 0.9999 | Fail to reject |
| Rcads46 | 0.020549 | 3 | 0.9992 | Fail to reject |
| Rcads47 | 0.017515 | 3 | 0.9994 | Fail to reject |
| df: Degree of freedom | | | | |

Supplementary Table S5. Results of the Mann Whitney Test for distributional properties between original and synthetic dataset (1: 4)

| **Questions** | **p-value** |
| --- | --- |
| rcads01 | 0.66 |
| rcads02 | 0.69 |
| rcads03 | 0.74 |
| rcads04 | 0.68 |
| rcads05 | 0.5 |
| rcads06 | 0.69 |
| rcads07 | 0.75 |
| rcads08 | 0.97 |
| rcads09 | 0.91 |
| rcads10 | 0.61 |
| rcads11 | 0.42 |
| rcads12 | 0.87 |
| rcads13 | 0.75 |
| rcads14 | 0.78 |
| rcads15 | 0.96 |
| rcads16 | 0.65 |
| rcads17 | 0.99 |
| rcads18 | 0.68 |
| rcads19 | 0.94 |
| rcads20 | 0.7 |
| rcads21 | 0.73 |
| rcads22 | 0.82 |
| rcads23 | 0.82 |
| rcads24 | 0.58 |
| rcads25 | 0.82 |
| rcads26 | 0.68 |
| rcads27 | 0.47 |
| rcads28 | 0.74 |
| rcads29 | 0.77 |
| rcads30 | 0.68 |
| rcads31 | 0.69 |
| rcads32 | 0.91 |
| rcads33 | 0.95 |
| rcads34 | 0.67 |
| rcads35 | 0.68 |
| rcads36 | 0.64 |
| rcads37 | 0.97 |
| rcads38 | 0.85 |
| rcads39 | 0.64 |
| rcads40 | 0.9 |
| rcads41 | 0.46 |
| rcads42 | 0.77 |
| rcads43 | 0.85 |
| rcads44 | 0.99 |
| rcads45 | 1 |
| rcads46 | 0.81 |
| rcads47 | 0.9 |
